# Cybersicherheit für Staat, Wirtschaft und Gesellschaft

**DOI:** 10.1007/s12399-023-00936-w

**Published:** 2023-02-22

**Authors:** Tobias Alexander Möller

**Affiliations:** Bundesamt für Sicherheit in der Informationstechnik (BSI), Godesberger Allee 185–189, 53175 Bonn, Deutschland

**Keywords:** Cybersicherheit, Bedrohungslage, Cybercrime, Ransomware, BSI, Cybersecurity, Cyber threats, Cybercrime, Ransomware, BSI

## Abstract

Der Beitrag fokussiert sich auf die unterschiedlichen Bedrohungen für Deutschland im Cyberraum und die Abwehr dieser Gefahren. Er thematisiert die gegenwärtige Bedrohungslage im Cyberraum durch eine praxisnahe Betrachtung von Akteuren und Methoden sowie die Entwicklungen in den Bereichen Ransomware und Advanced Persistent Threat. Zudem beleuchtet der Beitrag das Bundesamt für Sicherheit in der Informationstechnik als zentralen Akteur in der deutschen Cybersicherheit.

## Abstract (en)

The article focuses on the various threats to Germany in cyberspace as well as on the defense against these threats. The current threat situation in cyberspace is discussed through a practical examination of actors and methods as well as developments in the areas of ransomware and Advanced Persistent Threats. The article furthermore introduces the Federal Office for Information Security, which functions as a central actor in German cybersecurity.

## Einleitung

Am 8. Mai 2021 bestätigt das US-Unternehmen Colonial Pipeline Company, gemäß öffentlicher Darstellung der größte Pipeline-System-Betreiber für raffinierte Produkte in den USA, dass es einen Cyberangriff auf seine IT-Infrastruktur gegeben habe (Abrams 2021). Ein Ransomware^1^-Angriff hatte das Verwaltungsnetz des Unternehmens getroffen, woraufhin dieses abgeschaltet wurde. Überdies entschied das Unternehmen – als vorbeugende Sicherheitsmaßnahme – den Betrieb einer seiner Pipelines einzustellen (Sanger et al. 2021). Diese Nichtverfügbarkeit der Anlage führte anschließend zu regionalen Engpässen bei Treibstoffen sowie einer Verunsicherung in der Bevölkerung (Krauss et al. 2021). Lange Warteschlangen an den Tankstellen sowie ein Preisanstieg bei Treibstoffen waren die Folge. Das Federal Bureau of Investigation (FBI) teilte später mit, dass der Angriff mittels einer Ransomware namens Darkside durchgeführt worden sei (FBI 2021).

Einerseits führt der hier kurz skizzierte Vorfall das enorme Schadpotential eines erfolgreichen Ransomware-Angriffs vor Augen: Eine kritische Dienstleistung wird massiv beeinträchtigt. Andererseits zeigen sich bei diesem Vorfall nicht nur die unmittelbaren Auswirkungen eines Cyberangriffs, wie die Nichtverfügbarkeit von Waren oder Dienstleistungen, sondern vielmehr auch, dass darüber hinaus weitere Konsequenzen daraus resultieren können, die einen immensen politischen sowie öffentlichkeitswirksamen Schaden verursachen. Allein die Wahrnehmung der Bevölkerung, dass eine kritische Dienstleistung durch einen Cyberangriff eingeschränkt worden sein könnte, kann zu Unsicherheit und Unruhe in der Bevölkerung führen.

Wir leben im Zeitalter der Digitalisierung: Wir kaufen Waren digital, tätigen Banküberweisungen online und pflegen unsere Netzwerke mittels sozialer Medien. Informationstechnik^2^ in all ihren Formen ist ein selbstverständlicher Bestandteil unseres Lebens geworden. Durch die zunehmende digitale Vernetzung erschließen sich neue Wertschöpfungsmöglichkeiten und Annehmlichkeiten im Alltag. Hierbei ist es von Beginn an unabdingbar, bei dem Thema Digitalisierung auch das Thema Informationssicherheit mitzudenken, da auch unterschiedlichste Bedrohungsakteur*innen Informationstechnik zur Durchführung und Erlangung ihrer Ziele nutzen. Singer und Friedman (2014, S. 85) stellen fest: „As information technology grows more pervasive, however, it becomes harder to find crimes that don’t have a digital component“.

Der nachfolgende Beitrag hat zum Ziel, ein besseres, insbesondere praxisnahes Verständnis, für die aktuelle Bedrohungslage im Cyberraum zu schaffen, die letztendlich alle Nutzer*innen von Informationstechnik betrifft. Hierzu werden exemplarisch in Kapitel 2 sowohl die aktuelle Cybersicherheitslage als auch relevante Bedrohungsakteur*innen und genutzte Angriffsformen im Cyberraum betrachtet. Den Bedrohungen im Cyberraum stehen Akteur*innen aus den Bereichen der Prävention, Detektion und Reaktion gegenüber. Zentraler Akteur in Deutschland ist hier das Bundesamt für Sicherheit in der Informationstechnik (BSI) als die Cybersicherheitsbehörde des Bundes. Die Aufgaben, Befugnisse und Angebote des BSI sind Bestandteil des Kapitels 3. In Kapitel 4 werden abschließend die zuvor erörterten Beispiele, Trends und Aspekte zusammengeführt sowie ein Ausblick auf weitere mögliche Entwicklungslinien im Bereich der Cybersicherheit gegeben.

## Bedrohungslage

Das BSI beobachtet, analysiert und bewertet fortlaufend die Gefährdungslage der Cybersicherheit in Deutschland. Diese wird nachfolgend dargestellt.

### Allgemeine Cybersicherheitslage

Eine ohnehin seit Jahren angespannte Bedrohungslage im Cyberraum (vgl. u. a. BSI 2017, 2018, 2019, 2020a) trifft seit 2020 nunmehr auf neue Herausforderungen für Staat, Wirtschaft und Gesellschaft, da die Covid-19-Pandemie zu einer rapiden, gar sprunghaften Digitalisierung in verschiedensten Arbeits- und Lebensbereichen führte. Um die Arbeit aus der Ferne zu ermöglichen, etablierten Organisationen und Unternehmen kurzerhand Fernwartungs- und VPN-Zugänge, um schnellstmöglich eine Remote-Arbeitsfähigkeit der Arbeitnehmer*innen herzustellen. Falsch konfigurierte und nicht ausreichend abgesicherte Fernwartungs- und VPN-Zugänge sind jedoch auch bevorzugte Einfallstore für Bedrohungsakteur*innen im Cyberraum. Während erfolgreiche Cyberangriffe schon unter „normalen“ Umständen schwerwiegende Folgen für die Betroffenen haben, können diese während der Covid-19-Pandemie und infolgedessen einer erhöhten Abhängigkeit von Informationstechnik existenzbedrohend für Organisationen und Unternehmen sein.

Seit 2022 kommt der russische Angriffskrieg gegen die Ukraine hinzu, der auch im Cyberraum beobachtbare Effekte zeigt. Bereits zuvor finanziell orientierte Cyberkriminelle sowie Hacktivist*innen schlagen sich auf die jeweiligen Seiten der Kriegsparteien (Anonymous 2022; Greig 2022). Zudem rief die ukrainische Regierung zur Gründung der IT Army of Ukraine auf, die aus Freiwilligen sowie Sympathisant*innen besteht und zum Schutz der Kritischen Infrastrukturen des Landes sowie zur Durchführung von Cyberangriffen gegen das russische Militär beitragen soll (Pearson 2022; Schectman und Bing 2022).

Der Cyberraum charakterisiert sich allgemein durch eine gewisse Grenzenlosigkeit, sodass Cyberangriffe gleichermaßen in allen Staaten, auch in Deutschland, ihre Wirkung entfalten oder Kollateralschäden fordern können. Unternehmensnetze sind heutzutage weltweit miteinander verbunden und können bei einem Vorfall an einem Standort auch die Netze anderer Standorte beeinträchtigen. Cyberangriffe können so auch über die Ukraine oder Russland hinauswirken.

Deutsche Einrichtungen und Unternehmen geraten auch direkt in das Visier von Hacktivist*innen. Beispielsweise griff eine Gruppierung namens Killnet im April 2022 deutsche Ziele mittels Distributed Denial of Service (DDoS)-Angriffen^3^ an, wodurch zeitweise einige Webseiten nicht mehr erreichbar waren (Gebauer et al. 2022). Diese technisch vergleichsweise wenig anspruchsvollen Angriffe führen zwar kurzzeitig zur einer Überlastung der angegriffenen Systeme oder Webseiten, können bei bestehenden Schutzmaßnahmen jedoch schnell mitigiert werden. Insgesamt kam es in Deutschland bislang nur zu einzelnen Cybersicherheitsvorfällen im Zusammenhang mit dem russischen Angriffskrieg gegen die Ukraine, die nur geringe Auswirkungen für die Betroffenen nach sich zogen (BSI 2022). Eine zwischenzeitlich befürchtete, massive Ausbreitung von Cyberangriffen, auch auf deutsche Netze, blieb bislang aus.^4^ Dennoch ist die gegenwärtige Lage weiterhin äußerst volatil und kann sich sekündlich ändern. Daher beobachtet das BSI auch weiterhin die Lage der Informationssicherheit im Hinblick auf den russischen Angriffskrieg gegen die Ukraine und tauscht sich stetig mit seinen nationalen und internationalen Partnerbehörden aus (BSI 2022).

Zum gegenwärtigen Zeitpunkt ist eine Vielzahl an unterschiedlichen Akteur*innen feststellbar, die sowohl die Kapazitäten als auch den Willen besitzen, Staat, Wirtschaft und Gesellschaft unter Einsatz von Cyberangriffen unmittelbar und mittelbar zu schädigen, zu stören oder zu sabotieren. Zwar unterscheiden sich diese zum Teil deutlich in ihren Motivationen für Cyberangriffe, doch greifen sie immer häufiger auf die gleichen Werkzeuge und Vorgehensweisen zurück. Der nachfolgende Abschnitt wird exemplarisch auf drei häufige Arten von Bedrohungsakteur*innen eingehen.

### Akteur*innen

Vielfältige Akteur*innen greifen aufgrund der Schnelligkeit, Anonymität, Ort- und Grenzenlosigkeit sowie infolge verhältnismäßig geringer Kosten auf Cyberangriffe zurück. Während Spionage, Sabotage sowie weitere Straftaten früher durch Personen(-gruppen) in rein physischer Form und vor Ort durchgeführt werden mussten, können Täter*innen heutzutage mittels Cyberangriffen aus der Ferne, mit einem gewissen „Sicherheitsabstand“, agieren. Statt physischer Gewalt greifen sie auf digitale Angriffsmethoden zurück und bedrohen die Verfügbarkeit, die Vertraulichkeit, die Integrität und die Authentizität der Informationstechnik.

Gemäß öffentlicher Quellen (Mandiant o.J.) nutzt vermutlich eine kleine Anzahl von Staaten zielgerichtete Cyberangriffe, um Zielstaaten zu schädigen oder strategische Informationen zu erlangen. Dabei greifen sie in ihrem operativen Handeln teilweise auch auf nichtstaatliche Akteur*innen, beispielsweise aus der organisierten Kriminalität, zurück (Canadian Centre for Cyber Security 2021, S. 3).

Zielgerichtete Cyberangriffe werden auch im Kontext hybrider Kampagnen eingesetzt, in denen verschiedene Maßnahmen orchestriert werden, um ein Zielland zu destabilisieren (BSI 2021b, S. 37–38). Staatliche Akteure können meist auf eine Fülle von Ressourcen (u. a. monetär und technisch) zurückgreifen, um komplexe Cyberangriffe mit hochentwickelten Methoden, beispielsweise Spionageangriffen, durchzuführen (Canadian Centre for Cyber Security 2021, S. 3).

Auf Seiten der finanziell motivierten Täter*innen (Cyberkriminelle) ist in den letzten Jahren eine Veränderung hin zu einer stetigen Professionalisierung festzustellen. Die früher sichtbaren technischen Unterschiede – im Vergleich zu staatlichen Akteuren – verschwimmen zunehmend. Im Rahmen ihrer Angriffe setzen Cyberkriminelle unterschiedlichste Angriffsmethoden ein, u. a. Ransomware, DDoS-Angriffe sowie Phishing, also das gezielte „fischen“ nach Zugangsdaten eines Opfers. Abhängig von der Professionalität und der zur Verfügung stehenden Ressourcen der Cyberkriminellen sowie der bestehenden Vorsorge- und Schutzmaßnahmen des Angriffsziels können die entstehenden Schäden und Folgen für den Betroffenen immens, gar existenzbedrohend sein.

Neben staatlichen Bedrohungsakteuren und Cyberkriminellen, stellen auch Hacktivist*innen eine Bedrohung für die Cybersicherheit dar. Diese können durch öffentlichkeitswirksame Cyberangriffe für ihre Agenda und politische Überzeugung werben sowie Bevölkerungsgruppen, wie beispielsweise Minderheiten, einschüchtern. Der Krieg in der Ukraine beweist einmal mehr, dass auch von hacktivistischen Gruppierungen eine aktive Gefahr für die Cybersicherheit ausgehen kann.

Nachdem nun eine kurze Übersicht zu unterschiedlichen Arten von Bedrohungsakteur*innen gegeben wurde, wird der nachfolgende Exkurs zwei prominente Angriffsformen näher erläutern.

### Exkurs: Angriffsformen

Es existiert eine Vielzahl unterschiedlichster Angriffsformen und Schadsoftwarearten. Der nachfolgende Exkurs richtet seinen Fokus auf die Themen Ransomware und Advanced Persistent Threats (APTs).

*Ransomware* Ransomware gilt derzeit als eine der größten Bedrohungen für die Sicherheit der Informationstechnik von Staat, Wirtschaft und Gesellschaft, da sie vollumfänglich im Netzwerk erreichbare Daten und Systeme eines Betroffenen verschlüsseln kann. Insbesondere Cyberkriminelle nutzen Ransomware, um Lösegelder zu erpressen. Mögliche Szenarien reichen von einem Ausfall einzelner Arbeitsplätze bis hin zum Stillstand ganzer Produktionsstandorte.

Ransomware-Angriffe können ungezielt-zufällig erfolgen, beispielsweise im Rahmen einer breit angelegten Kampagne, die versucht, möglichst viele Opfer zu kompromittieren, um anschließend eine Vielzahl von Betroffenen zu erpressen. Andere Cyberkriminelle konzentrieren ihre Angriffe hingegen auf besonders finanzstarke Opfer, beispielsweise große Unternehmen, um nach einem erfolgten Angriff höchstmögliche Lösegeldsummen erpressen zu können. Dieses Phänomen wird auch als *Big Game Hunting* bezeichnet (BSI 2021b, S. 12).

Cyberkriminelle bedienen sich unterschiedlicher initialer Angriffsmethoden, um ihre potentiellen Opfer mit Ransomware anzugreifen. Hierzu zählen unter anderem Schwachstellen in Systemen infolge einer fehlerhaft konfigurierten oder nicht aktualisierten Software sowie bekannt gewordene Schwachstellen in Softwareprodukten selbst. Auch versandte maliziöse E‑Mails, bei denen unter Umständen auch hoch entwickelte Social-Engineering^5^-Techniken angewandt werden, können als Angriffsvektor für Cyberkriminelle dienen (BSI 2021a, S. 1). Um eine schadhafte E‑Mail möglichst authentisch aussehen zu lassen, wird die Absenderadresse der E‑Mail gefälscht sowie – sofern vorliegend – auf weitere ausgespähte E‑Mail-Inhalte mit dem Kommunikationspartner referenziert. Ein bekannter Absender, eine bekannte Betreffzeile oder gar zitierte E‑Mail-Inhalte erhöhen die Glaubwürdigkeit einer E‑Mail, können Empfänger*innen in falscher Sicherheit wiegen und erhöhen damit die Wahrscheinlichkeit, dass Empfänger*innen einen maliziösen Link oder ein Köder-Dokument öffnet, herunterlädt oder gar schädliche Inhalte ausführt.

Im Anschluss an die initiale Infektion des Betroffenen versuchen sich die Täter*innen lateral im jeweiligen Netzwerk auszubreiten. Dafür nutzen die Cyberkriminellen unterschiedliche Mittel, beispielsweise die Nutzung von Zugangsdaten oder das Nachladen weiterer Schadsoftware. Anschließend wird entweder mit der Verschlüsselung der Systeme und Nutzerdaten (u. a. Office‑, Bild‑, Ton- und Videodateien sowie ganze Datenbanken) des Betroffenen mittels einer Ransomware begonnen oder es werden zuvor noch Daten ausgeleitet, die im späteren Verlauf als Beweis für eine erfolgreiche Kompromittierung oder als weiteres Druckmittel in den Lösegeldverhandlungen dienen (BSI 2021a, S. 2–3).

Sobald eine Verschlüsselung erfolgte, kontaktieren die Täter*innen den Betroffenen, häufig mittels einer hinterlassenen Nachricht auf den kompromittierten Systemen. Die Täter*innen stellen dem Betroffenen eine Entschlüsselungslösung in Aussicht, sofern die Lösegeldforderung, meist in Form von Kryptowährungen wie Bitcoin oder Monero, bedient wird. Die Abwicklung mittels Kryptowährungen erschwert aufgrund ihrer Anonymität eine Straf- sowie Geldverfolgung durch Behörden. Cyberkriminelle setzen Betroffene in den Lösegeldverhandlungen bewusst unter Druck, unter anderem durch die Setzung kurzer Zahlungsfristen, um schnellstmöglich ein Lösegeld zu erpressen und einer analogen Wiederherstellung der Systeme durch den Betroffenen, beispielsweise mittels funktionierender Back-Ups oder Entschlüsselung der Ransomware, zuvorzukommen.

Es lässt sich beobachten, dass erfolgreiche Angriffsmethoden zeitnah durch andere Cyberkriminelle adaptiert werden. Ein Beispiel hierfür ist das Phänomen der *Double Extortion*: Hierbei leiten Cyberkriminelle zunächst Daten eines Opfers aus, bevor sie die Systeme mittels Ransomware verschlüsseln. Die so erbeuteten Daten dienen in den anschließenden Löse- beziehungsweise Schweigegeldverhandlungen als zusätzliches Druckmittel, um Betroffene zu einer Zahlung zu nötigen, da anderenfalls die Daten auf dedizierten Veröffentlichungswebseiten der Cyberkriminellen publiziert werden. Beobachtungen deuten darauf hin, dass allein im Zeitraum 2020 bis 2021 die Anzahl solcher Veröffentlichungsseiten um rund 360 % angestiegen ist (BSI 2021b, S. 13). Auf diesen Webseiten findet sich meist eine Vielzahl an betroffenen Organisationen, die nicht mit den Cyberkriminellen kooperierten und nun öffentlich bloßgestellt werden.

Diese kombinierten Erpressungsmethoden sind zuletzt gängige Praxis von Cyberkriminellen geworden. Betroffene Daten müssen fortan als kompromittiert, beziehungsweise verloren, angesehen werden, da sie jederzeit durch die Täter*innen veröffentlicht, verkauft oder vervielfältigt werden können (BSI 2021b, S. 14). Die Folgen eines derartigen Ransomware-Angriffs können für Betroffene existenzbedrohend sein: Eine Veröffentlichung von Daten kann massive Reputationsverluste bedeuten, beispielsweise gegenüber Kunden. Auch der Verlust von elementaren Geschäftsgeheimnissen sowie die Veröffentlichung diskreditierender Daten sind mögliche negative Folgen.

Die beachtlichen Folgen eines Ransomware-Angriffs zeigen sich am Beispiel eines deutschen Universitätsklinikums. Nachdem am 10. September 2020 ein Ransomware-Angriff erkannt wurde, mussten Verbindungen zum Internet getrennt und Systeme heruntergefahren werden, um das Schadensmaß einzugrenzen. Die Erreichbarkeit des Klinikums war daraufhin massiv eingeschränkt. Eine Behandlung stationärer Patient*innen konnte zwar weiterhin gewährleistet werden, jedoch war es nicht mehr möglich, neue Patient*innen aufzunehmen. Das Klinikum reagierte zwar unmittelbar auf den Vorfall und wurde durch das BSI im gesamten Zeitraum unterstützt, musste sich jedoch aufgrund der Schwere des Vorfalls für einen Zeitraum von 13 Tagen von der Notfallversorgung abmelden (BSI 2021b, S. 15).

*Advanced Persistent Threats* Unter dem Begriff Advanced Persistent Threats fallen zielgerichtete Cyberangriffe, die sich im Vergleich zu anderen Angriffsformen durch einen hohen Ressourceneinsatz und erhebliche technische Fähigkeiten auf Seiten der Angreifer*innen charakterisieren und daher häufig von staatlichen Akteuren ausgehen. Eine konkrete Attribution von APT-Gruppierungen zu Staaten erfolgt dabei nicht durch das BSI. Im Vergleich zu dem Vorgehen von Cyberkriminellen unterscheidet sich die Vorgehensweise der Angreifer*innen sowohl in der Motivation als auch teilweise in dem Vorgehen der Täter*innen (Steffens 2018, S. 7–9).

APT-Angriffe dienen häufig der Durchführung von Spionage-Operationen, also der Erlangung von Wissen über den Betroffenen, sowie zur Sabotage, also der gezielten Schädigung eines Opfers. Angriffe dieser Art betreffen, je nach Missionsziel, unterschiedlichste Akteur*innen aus Staat, Wirtschaft und Gesellschaft, unter anderem Regierungseinrichtungen, für Angreifer*innen relevante Unternehmen, Kritische Infrastrukturen sowie Akteur*innen aus dem (vor-)politischen Raum.

APTs charakterisieren sich durch eine langfristige Planung des Angriffs sowie einen hohen Aufwand, um möglichst unerkannt in Zielsysteme einzudringen und das jeweilige Missionsziel zu erreichen. Als initialen Angriffsvektor nutzen APT-Gruppen häufig komplexe Vulnerabilitäten oder den Software-Herstellern bislang unbekannte Schwachstellen, sogenannte Zero-Day-Exploits. Andere Gruppen hingegen versenden zunächst schadhafte Mailanhänge oder Links, die Schadsoftware nachladen oder Zugangsdaten erbeuten. Hierzu nutzen APT-Gruppen teilweise exponierte internationale Veranstaltungen, wie zum Beispiel Regierungsgipfel, um mittels Phishing-Angriffen Zugangsdaten von hochrangigen Regierungsvertreter*innen zu erbeuten.

Sobald es APT-Gruppen gelingt, Zugriff auf ein Netz zu gewinnen, beginnen sie, ihre Zugriffsmöglichkeiten und -rechte auf weitere Systeme auszuweiten sowie Hintertüren in die Systeme einzubauen, um bei einem Bekanntwerden der initialen Kompromittierung weitere Hintereingänge in die Systeme nutzen zu können. APT-Gruppen nutzen unterschiedliche Techniken und Angriffswerkzeuge, die sowohl öffentlich verfügbare Werkzeuge beinhalten, wie beispielsweise frei erwerbliche Software zur Durchführung von Penetrationstests^6^, oder aber selbstentwickelte, hochkomplexe und gruppenspezifische Schadsoftware.

Die Täter*innen versuchen ihre Angriffe stets möglichst unbemerkt durchzuführen, um nicht durch etwaige Sicherheitssysteme detektiert zu werden. Auch eine zielgerichtete Verschleierung ihrer Aktivitäten sowie *False-Flag*-Methoden (Shevchenko und Nish 2017) werden genutzt, um eine spätere forensische Untersuchung der Systeme zu erschweren oder gar zu täuschen.

Eine Kompromittierung kann zudem durch eine infiltrierte Software-Supply-Chain erfolgen. APT-Gruppen greifen hierfür die Hersteller von Software an und implementieren Schadcode in legitimer Software oder in Updates. Die Tarnung als legitime Software ermöglicht den Täter*innen einerseits bestehende Detektionsmaßnahmen zu unterwandern und andererseits bei weit verbreiteten Softwareprodukten eine hohe Anzahl an Systemen und Netzwerken potentiell kompromittieren zu können (BSI 2021b, S. 29).

## Das Bundesamt für Sicherheit in der Informationstechnik (BSI)

Das BSI ist im Vergleich zu anderen Bundesämtern eine relativ junge Bundesoberbehörde im Geschäftsbereich des Bundesministeriums des Innern und für Heimat. Es nahm im Jahr 1991, infolge des am 17. Dezember 1990 verabschiedeten BSI-Errichtungsgesetzes, seine Arbeit auf.

Heute arbeiten mehr als 1400 Mitarbeitende an den deutschlandweiten Standorten des BSI. Hierzu zählen neben dem offiziellen Dienstsitz in Bonn ein weiterer Standort in Freital, ein Stützpunkt in Saarbrücken sowie weitere regionale Verbindungsstellen in Berlin, Bonn, Stuttgart, Wiesbaden und Hamburg.

Seit seiner Gründung entwickelt sich das BSI parallel zur Digitalisierung fortlaufend weiter und engagiert sich in allen relevanten Themen der Informationstechnik. Heute ist das BSI die Cybersicherheitsbehörde des Bundes und wesentlicher Gestalter einer sicheren Digitalisierung in Deutschland. Das BSI kooperiert hierbei national und international mit seinen Partnern und bietet unterschiedlichste Produkte, Dienstleistungen und Informationen für Staat, Wirtschaft und Gesellschaft in den Bereichen Prävention, Detektion und Reaktion an. Aufgrund der Vielzahl an gesetzlichen Aufgaben, Befugnissen und Angeboten des BSI kann nachfolgend nur ein exemplarischer Auszug dargestellt werden.

Die zentrale Zuständigkeit des BSI ist die Abwehr von Cyberangriffen auf die Regierungsnetze^7^ und die Bundesverwaltung. Hierzu zählt beispielsweise auf Seiten der Detektion die fortlaufende Beobachtung der Cybersicherheitslage im nationalen und internationalen Kontext, unter anderem durch das BSI-Lagezentrum, welches auch als zentrale Meldestelle für Cybersicherheitsvorfälle und -angriffe in Deutschland dient. Auch die Analyse und Bewertung bestehender Sicherheitsrisiken und Schwachstellen wird durch das BSI gewährleistet. Im Rahmen seiner gesetzlichen Zuständigkeiten und Befugnisse leistet das BSI überdies Unterstützung bei IT-Sicherheitsvorfällen durch das Computer Emergency Response Team (CERT-Bund), das sowohl präventive als auch reaktive Hilfe zur Verfügung stellt. Zudem kann das mobile Einsatzteam (Mobile Incident Response Team – MIRT) innerhalb kürzester Zeit bei der Aufrechterhaltung und Wiederherstellung von Systemen eines Betroffenen – direkt vor Ort – unterstützen. Allein im Zeitraum 2019 bis 2020 konnte das BSI deutschlandweit bei rund 70 IT-Sicherheitsvorfälle unterstützen (vgl. BSI 2020a, S. 71).

Ebenso obliegt dem BSI die Aufsicht über die Sicherheit in der Informationstechnik von Kritischen Infrastrukturen und digitalen Diensten (Gesetz über das Bundesamt für Sicherheit in der Informationstechnik BSIG). Gemäß BSIG gelten Organisationen und Einrichtungen als Kritische Infrastrukturen, wenn sie den Sektoren Energie, Informationstechnik und Telekommunikation, Transport und Verkehr, Gesundheit, Wasser, Ernährung, Finanz- und Versicherungswesen sowie Siedlungsabfallentsorgung angehören und zudem „von hoher Bedeutung für das Funktionieren des Gemeinwesens sind, weil durch ihren Ausfall oder ihre Beeinträchtigung erhebliche Versorgungsengpässe oder Gefährdungen für die öffentliche Sicherheit eintreten würden“ (BSIG § 2 Absatz 10). Eine nähere Bestimmung der Kritischen Infrastrukturen erfolgt zudem durch die Verordnung zur Bestimmung Kritischer Infrastrukturen nach dem BSI-Gesetz (BSI-Kritisverordnung - BSI-KritisV).

Eine Erhöhung der Resilienz des Wirtschaftsstandorts Deutschland vor Bedrohungen im Cyberraum ist für den Erfolg der Digitalisierung enorm wichtig. Daher gründete das BSI in Zusammenarbeit mit dem Bundesverband Informationswirtschaft, Telekommunikation und neue Medien e. V. (BITKOM) die Allianz für Cyber-Sicherheit (ACS). Das Netzwerk ermöglicht den Teilnehmenden einen Austausch von IT-Expertise und Anwendungserfahrungen. Es besteht mittlerweile seit 10 Jahren und verzeichnet 6479 teilnehmende Unternehmen und Institutionen – Tendenz weiter steigend (BSI o.J.c).

Der *IT-Grundschutz* des BSI, welcher seit dem Jahr 1994 seitens des BSI unterstützt und begleitet wird, gilt heute als präventives Standardwerk für ein ganzheitliches IT-Sicherheitsmanagement. Es enthält Anleitungen, Empfehlungen und Methoden zur Absicherung von eingesetzter Informationstechnik. Neben der technischen Ebene thematisiert der IT-Grundschutz auch infrastrukturelle, organisatorische und personelle Aspekte (BSI 2020b).

Das BSI unterstützt zudem Verbraucher*innen in der Risikobewertung von Technologien, Produkten, Dienstleistungen und Medienangeboten. Hierzu stellt es Informationen zu verschiedenen Themen zur Verfügung, bietet Awareness-Maßnahmen an und steht als Ansprechpartner mit einem Service Center zur Verfügung (BSI o.J.d).

Das BSI als Gestalter in der Digitalisierung identifiziert und begleitet darüber hinaus eine Vielzahl an Zukunftsthemen, Trends und Digitalisierungsprojekten. Beispiele sind der Mobilfunkstandard 5G/6G, unterschiedlichste Formen der Künstlichen Intelligenz und Quantentechnologie. In diesem Zusammenhang steht es auch im regelmäßigen Austausch mit internationalen Herstellern von Informations- und Kommunikationstechnik.

## Fazit

Die in diesem Beitrag exemplarisch beleuchteten Ereignisse, Bedrohungsakteur*innen sowie Angriffsformen im Cyberraum unterstreichen, dass die aktuelle Cybersicherheitslage angespannter ist denn je. Es lässt sich insgesamt gar von einer angespannten bis kritischen Cybersicherheitslage sprechen.

Die Zunahme des Homeoffice ist eine digitale Herausforderung, die nur bei gleichzeitiger Investition und Begleitung in und durch Cybersicherheit gelingen kann. Der Krieg in der Ukraine wird immer wieder auch durch Cyberangriffe begleitet und gefährdet damit die Cybersicherheit von deutschen Organisationen und Unternehmen. Wenngleich die Auswirkungen auf Deutschland bislang gering ausfielen, bleibt die Lage äußerst volatil. Welche langfristigen Folgen aus der aktuell starken Rolle von Hacktivist*innen in dem Krieg entstehen, ist bislang unbekannt und kann neue Herausforderungen für die Cybersicherheit bedeuten.

Die beobachtbare gesteigerte Qualität und Adaptionsfähigkeit der cyberkriminellen Akteur*innen erhöht zunehmend die Gefahr für Organisationen und Unternehmen, Opfer eines Angriffs zu werden. Die digitale Erpressung beweist sich als die zentrale Herausforderung für Betroffene. Erfolgreiche Cyberangriffe haben gleichzeitig immer häufiger fatale Folgen für die Betroffenen. Der Angriff auf eine Pipeline in den USA führte schnell zu drastischen Auswirkungen. Angriffe auf das Gesundheitswesen können eine Gefahr für Leib und Leben der Patient*innen bedeuten, sobald betriebsrelevante Systeme betroffen sind. Dabei ist heutzutage nicht mehr die Frage, ob solche Vorfälle wieder geschehen, sondern wann.

Eine erfolgreiche Digitalisierung kann daher nur gelingen, wenn diese ausreichend sicher gestaltet wird und als nationale, gemeinsame Aufgabe von Staat, Wirtschaft und Gesellschaft verstanden wird. Das BSI begleitet diese Prozesse als die Cybersicherheitsbehörde des Bundes und engagiert sich in den Feldern der Prävention, Detektion und Reaktion. Hierzu stellt es den unterschiedlichen Zielgruppen eine Vielzahl von Angeboten und Informationen zur Verfügung, ist Ansprechpartner für alle Themen der Informationssicherheit und kooperiert mit seinen nationalen und internationalen Partnern.

Am Ende liegt eine erfolgreiche Digitalisierung aber auch in den Händen der Nutzer*innen, die sich mit der sicheren Nutzung von Informationstechnik befassen, sich regelmäßig informieren und das eigene Nutzungsverhalten reflektieren sollten.

## Footnotes

1 Ransomware gilt als Schadprogramm, das die Verfügbarkeit von Daten mithilfe von Verschlüsselung einschränkt und nur gegen eine Lösegeldzahlung wieder freigibt (BSI 2021a, S. 1).

2 Im Verständnis des BSI umfasst Informationstechnik (IT) „alle technischen Mittel, die der Verarbeitung oder Übertragung von Informationen dienen. Zur Verarbeitung von Informationen gehören Erhebung, Erfassung, Nutzung, Speicherung, Übermittlung, programmgesteuerte Verarbeitung, interne Darstellung und die Ausgabe von Informationen“ (BSI o.J.a).

3 DDoS-Angriffe werden durch den Rückgriff auf eine große Anzahl von Computern oder Servern durchgeführt, um die Verfügbarkeit von Webseiten, Systemen oder Netzen einzuschränken (BSI o.J.a).

4 Stand Oktober 2022.

5 Bei Social-Engineering-Angriffen nutzen Täter*innen menschliche Schwächen aus, wie Neugier oder Angst, um Opfer zur eigenständigen Herausgabe von Daten oder zur Umsetzung gewisser Handlungen (Umgehung von Schutzmaßnahmen, die Installation schadhafter Software o. ä.) zu verleiten (BSI o.J.a).

6 Penetrationstests dienen zur Überprüfung der etablierten Sicherheitsmaßnahmen eines IT-Systems. Hierbei werden gezielt Angriffsversuche simuliert und anschließend ausgewertet (BSI o.J.a).

7 Gemäß des BSI wird mit „dem Begriff ‚Regierungsnetze‘ […] die Kommunikationsinfrastruktur für die zuverlässige und sichere Sprach- und Datenkommunikation zwischen den obersten Bundesbehörden und Verfassungsorganen in Deutschland bezeichnet“ (BSI o.J.b).
